# A Case of Acute Appendicitis With Mucocele, Serrated Adenoma, and Enterobius vermicularis as Underlying Etiologies

**DOI:** 10.7759/cureus.87628

**Published:** 2025-07-09

**Authors:** Nuno Carvalho, Madalena Trindade, Ana Lúcia Barreira, Brigitta Cismasiu, Carlos Luz

**Affiliations:** 1 Department of Surgery, Hospital Garcia de Orta, Almada, PRT; 2 Faculty of Medicine, University of Lisbon, Lisbon, PRT

**Keywords:** acute appendicitis, appendicular abscess, enterobius vermicularis, mucocele, serrated adenoma

## Abstract

A 58-year-old male presented to the emergency department with a clinical picture compatible with an appendicular abscess, which was resolved with parenteral antibiotics.

A colonoscopy showed mucus protruding through the appendicular lumen, compatible with the appendicular mucocele.

A laparoscopic appendectomy was performed. Histology revealed serrated adenoma, luminal *Enterobius vermicularis*,lymphoid hyperplasia, and features of chronic and acute inflammation of the appendix.

This case is unique, as all these features, i.e., mucocele, serrated adenoma, and *Enterobius vermicularis*, are simultaneously present in complicated acute appendicitis.

## Introduction

Acute appendicitis (AA) is the most common abdominal surgical emergency [1]. It is claimed that luminal obstruction by fecaliths in adults and lymphoid hyperplasia in pediatric patients is the initial event in AA. Other causes have been proposed, such as infection and luminal obstruction by tumors, or even an allergic component [2].

Appendicular abscess is the clinical presentation in 2-7% of cases of AA [3]. Tumors of the appendix are rarely depicted in the clinical picture of AA [4]. Appendicular mucocele can be an incidental finding or also be associated with AA [5]. When there is a clinical or imaging suspicion of appendicular mucocele, surgery is indicated due to the risk of present or future malignant transformation [5,6]. *Enterobius vermicularis* infection is the most common helminthic infection worldwide, but its role as an etiologic factor in AA is controversial [7].

Herein, we present a case of AA complicated by abscess. A colonoscopy showed mucus protruding through the appendicular orifice, which was compatible with appendicular mucocele. The appendix was surgically removed, and the specimen was pathologically diagnosed as containing *E. vermicularis*, serrated adenoma, chronic inflammatory infiltrate, and AA.

## Case presentation

The work has been reported in line with the CARE (Case Report) criteria [8].

A 58-year-old male presented in our emergency department with a two-week history of right lower quadrant abdominal pain, fever, and diarrhea for the last three to four days. His past medical history was unremarkable. On physical examination, the patient’s pulse rate was 84/min, blood pressure was 128/68 mmHg, and tympanic temperature was 38.5°C.

During the abdominal examination, a hard, mobile mass was felt in the right iliac fossa, with associated local tenderness. No other relevant findings were detected.

The patient presented with a normal leukocyte count and a marked increase in C-reactive protein, as shown in Table 1. Blood urea nitrogen, liver function tests, renal function, ionogram, and prothrombin time were normal.

**Table 1:** Blood analysis. Blood analytics: white blood cell count, absolute neutrophil count, and C-reactive protein.

Parameters	Patient values	Reference range
White blood cell count	10.4	4.5-11.0/mm3
Absolute neutrophil count	7.32	2.0-7.0/mm3
C-reactive protein	30.5	<0.1 mg/dL

**Figure 1:**
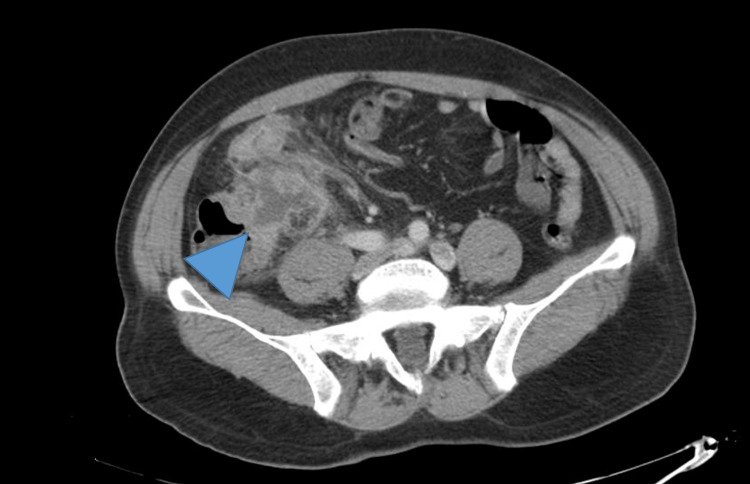
Periappendicular abscess. CT scan axial view showing periappendicular abscess (arrowhead).

**Figure 2:**
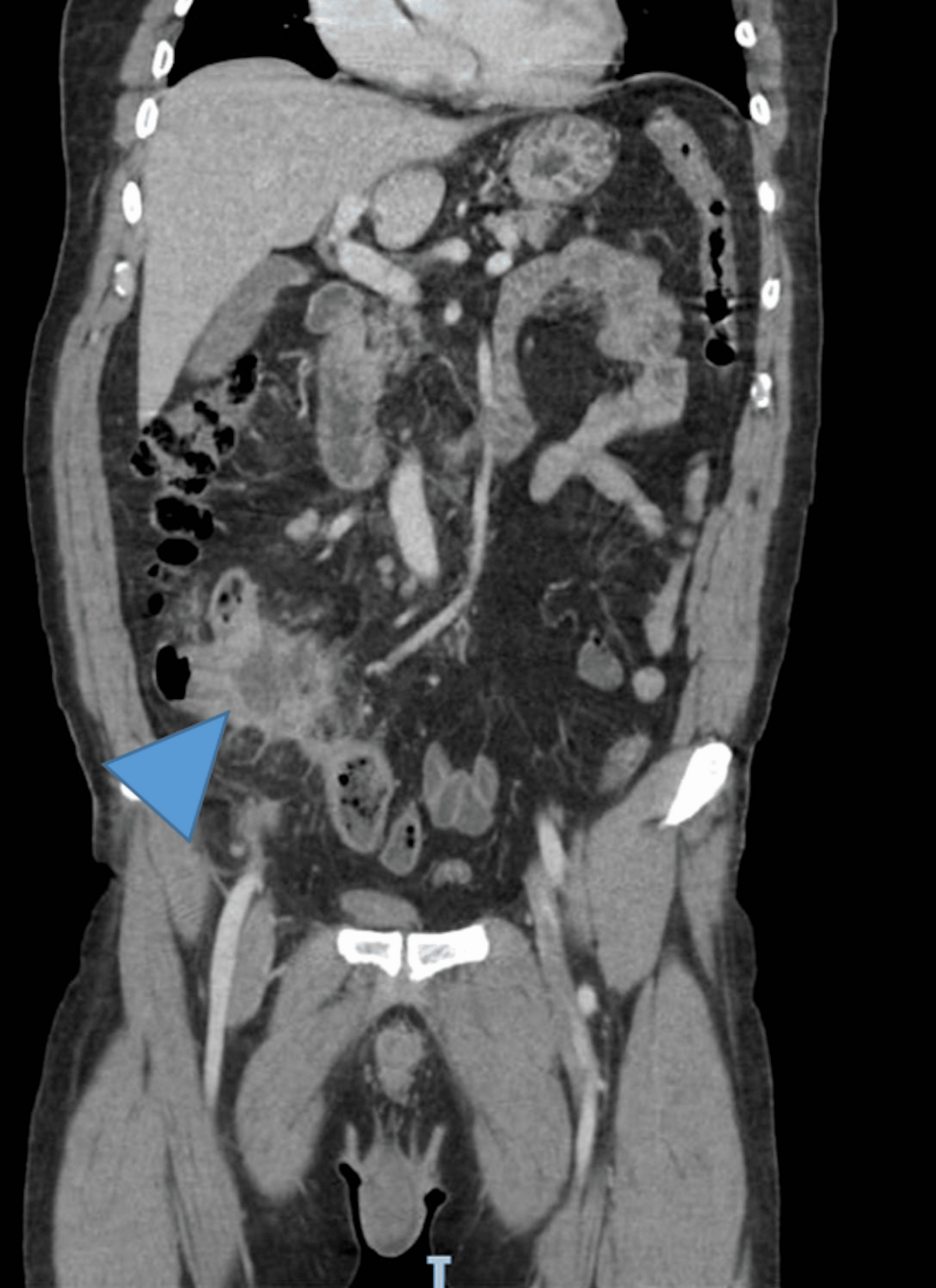
Periappendicular abscess. CT scan coronal view showing periappendicular abscess (arrowhead).

A CT scan was ordered and showed a ruptured appendix with a peri-appendicular abscess measuring 3.9 x 3.1 x 2.8 cm and lipomatous tissue densification in continuity. Parietal cecum and ileal last loop thickening were also present (Figures 1, 2).

A complicated appendicitis was diagnosed. Parenteral antimicrobial therapy consisting of 750 mg of cefuroxime every eight hours and 1000 mg of metronidazole every 12 hours was started.

After three days, the patient was discharged on oral antibiotics for the outpatient clinic due to a favorable clinical evolution: regression of pain and fever and a clear reduction of the palpable mass in the right iliac fossa. CRP and leucocyte count were low.

**Figure 3:**
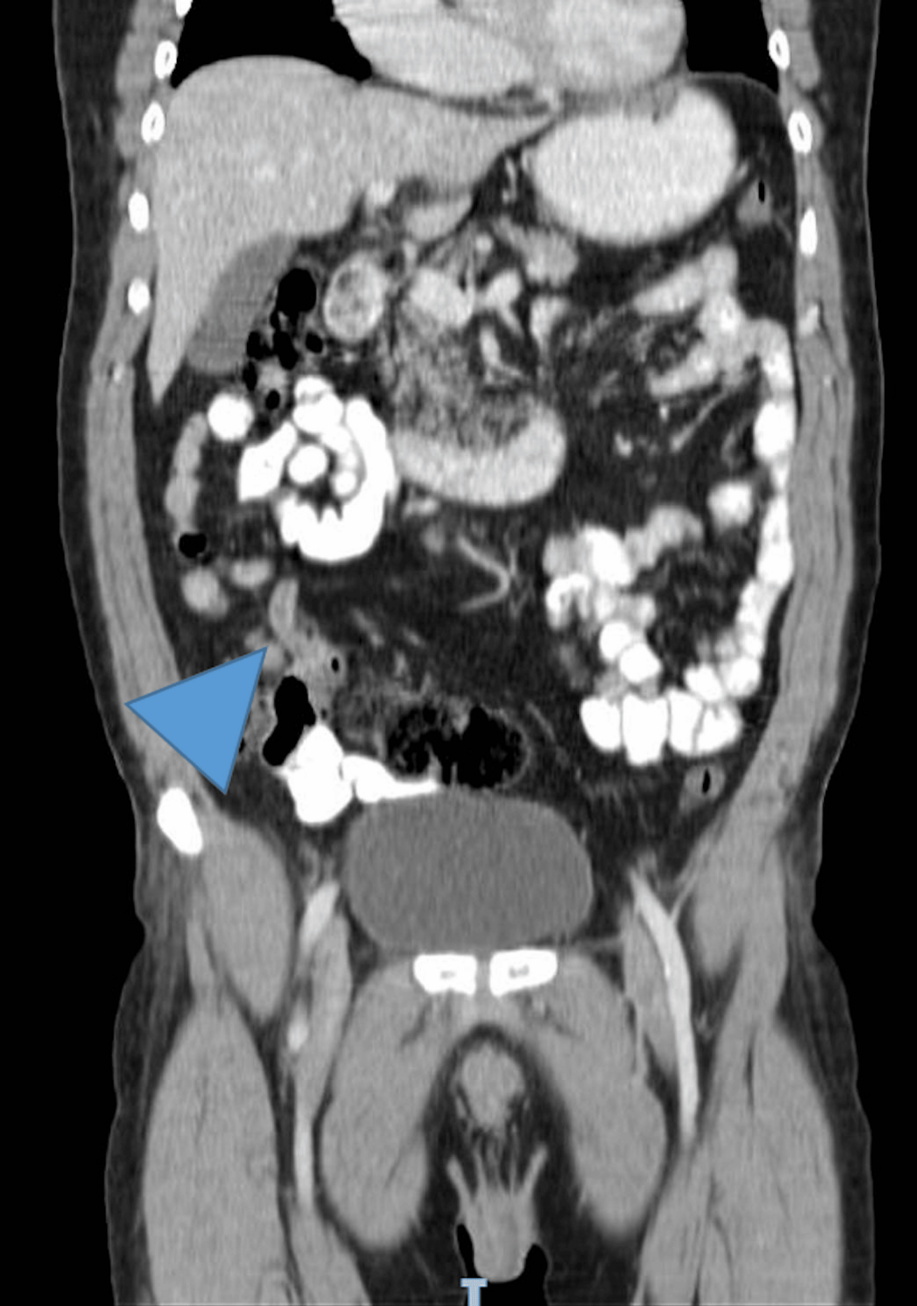
Thickened appendix. CT coronal view showing thickened appendix (arrowhead).

Six weeks later, a repeated CT scan showed a 12-13-mm-thickening appendix with an adjacent lymph node of 8 mm, but no other alterations (Figure 3).

**Figure 4:**
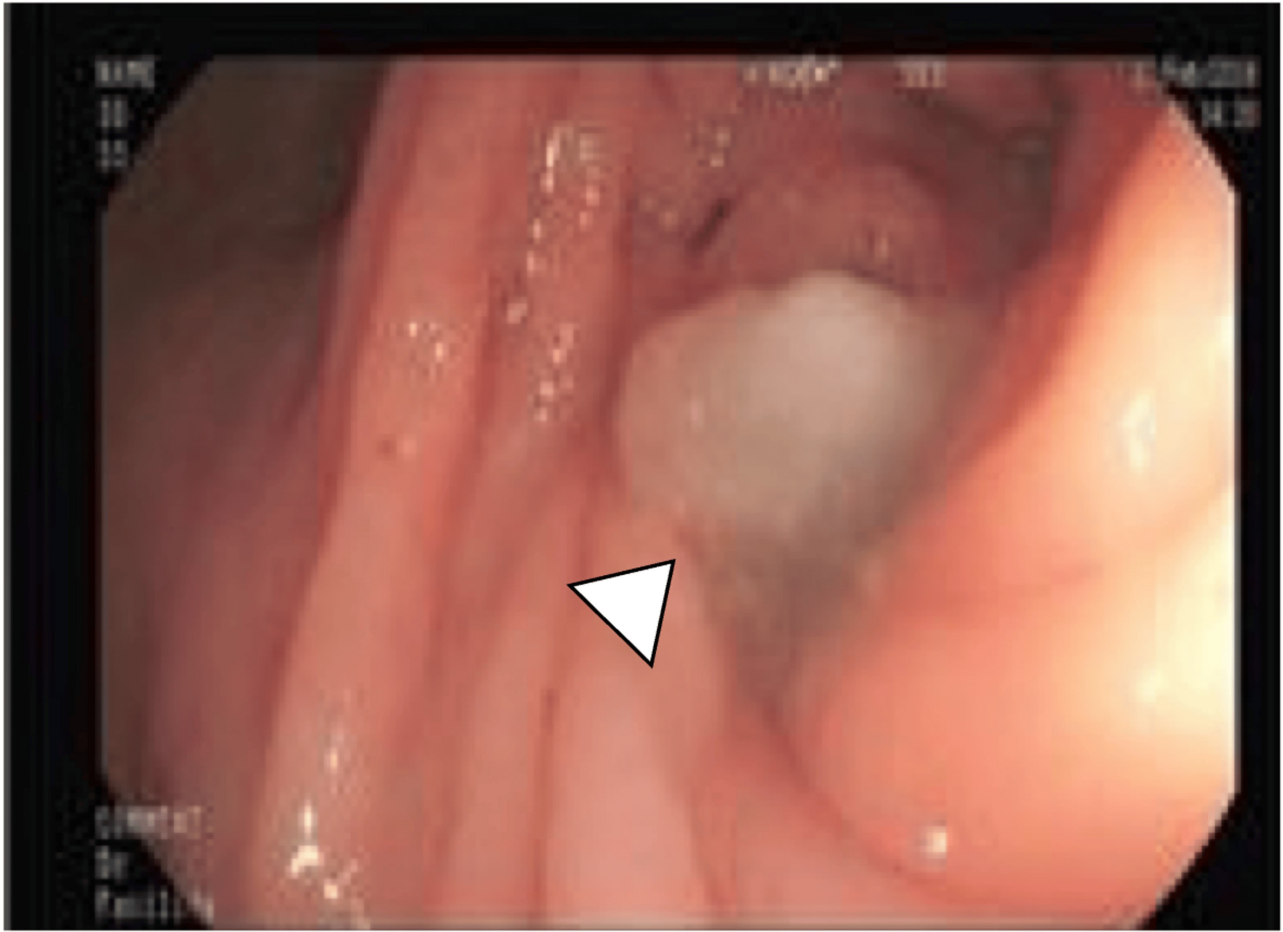
Appendicular lumen. Mucus fluid protruding through the appendicular lumen. Colonoscopy view (arrowhead).

A colonoscopy was ordered, which showed mucus fluid protruding through the appendicular lumen, being compatible with an appendicular mucocele (Figure 4).

Surgery was proposed due to the clinical diagnosis of appendicular mucocele [5]. At surgery, no pathologic process was found in the appendix, and the lymph nodes were not increased in size. An uneventful laparoscopic appendectomy was performed.

**Figure 5:**
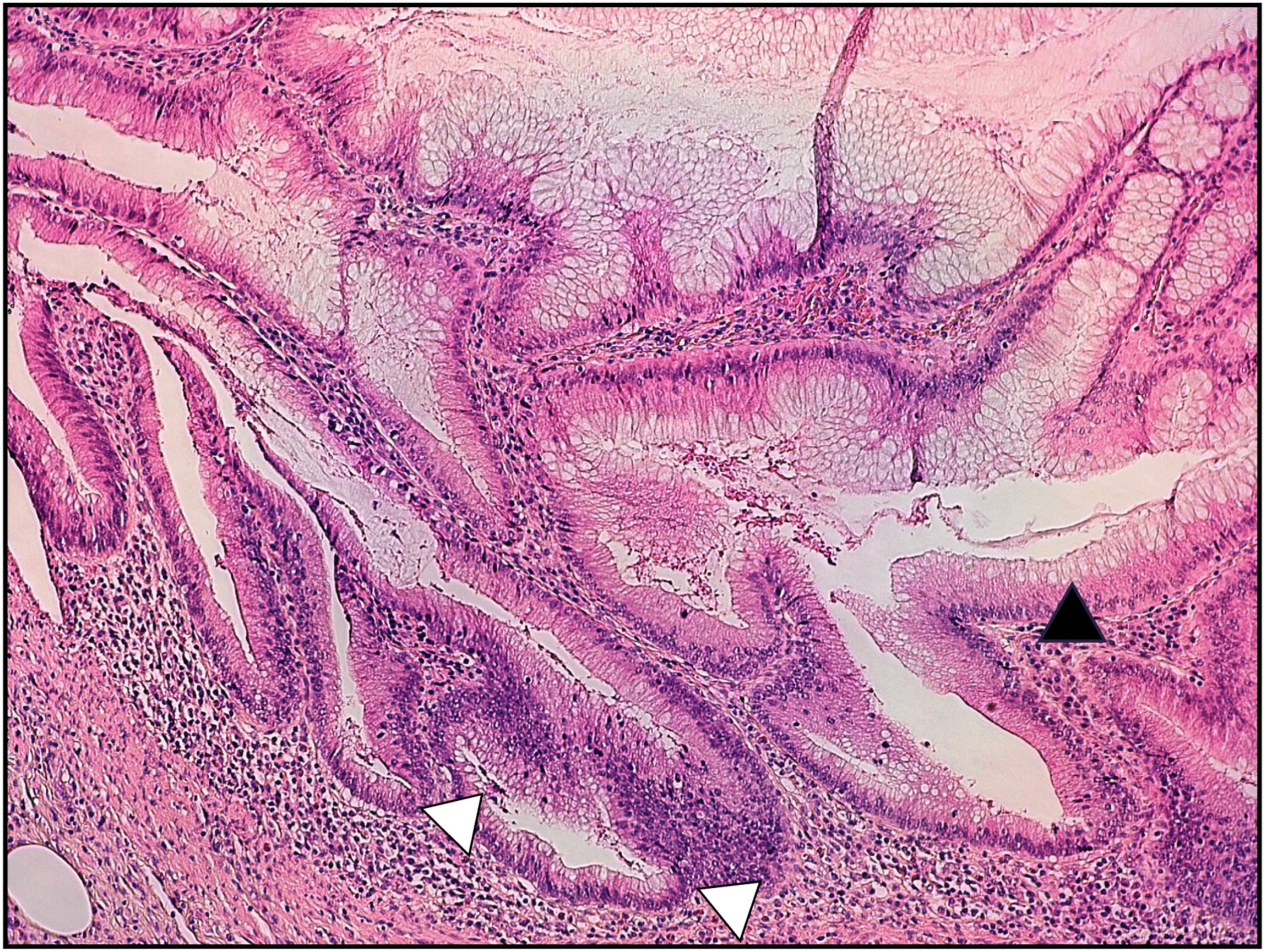
Sessile serrated adenoma. Sessile serrated adenoma of the appendix. Increased number of hyperplastic goblet cells in serrated crypts (black arrowhead). Basal dilation can be seen (white arrowheads). Courtesy: Daniel Pinto, MD, Pathologist.

**Figure 6:**
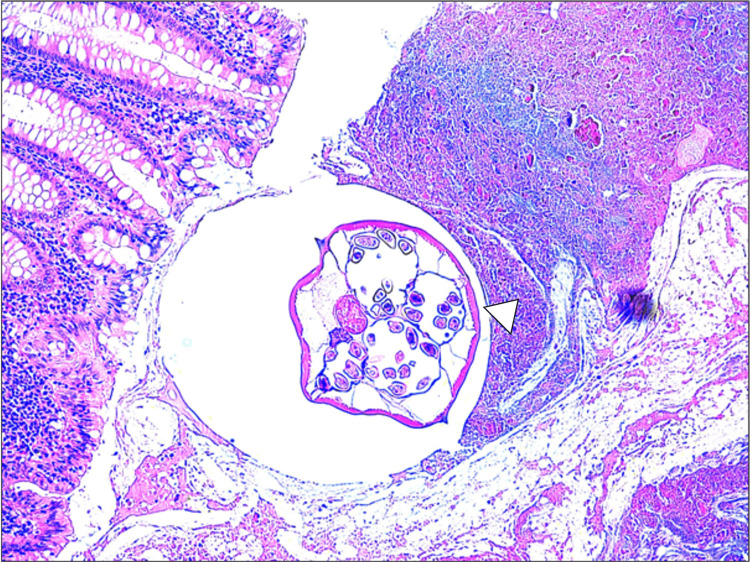
Enterobius vermicularis. Hematoxylin & eosin: Enterobius vermicularis. Cross-section of the worm (arrowhead). Courtesy: Daniel Pinto, MD, Pathologist.

Histology revealed lymphoid hyperplasia and a serrated adenoma without dysplasia (Figure 5). The appendicular lumen contained parasites with features compatible with *Enterobius vermicularis* (Figure 6). No mucus was present in the appendicular lumen.

The muscular propria was infiltrated with neutrophils. The final diagnosis was acute appendicitis, *Enterobius vermicularis* parasitosis, and appendicular serrated adenoma.

Two doses of albendazole were prescribed. Six years after the surgery, the patient is doing well with no complaints.

## Discussion

Appendicitis is the leading cause of abdominal emergency surgery. The etiology of AA is not understood, and several causes have been proposed [2]. Between 2% and 6% of patients with AA have an appendiceal mass, which is often described as an inflammatory phlegmon or abscess [9].

Conservative management and postponed appendectomy have been the traditional management for appendicular abscess [2,9]. Ultrasound or computed tomography-guided percutaneous drainage is conducted when conservative treatment fails. Surgical intervention is performed when the patient's condition does not improve [3]. The World Society of Emergency Surgery (WSES) Jerusalem guidelines recommend laparoscopic appendectomy [10]. The beneficial role of interval appendectomy for AA is still controversial, as the risk of recurrence is relatively small [3,10]. However, it may have some advantages, such as providing a definite diagnosis or ruling out an underlying malignancy.

In our case, the clinical evolution under conservative treatment was favorable and allowed patient discharge after three days.

Patients with appendiceal mass who are submitted to conservative treatment should undergo colonoscopy or barium enema to detect any underlying diseases and to rule out coexistent colorectal cancer [11].

There is no consensus regarding the right time to perform such investigations. It is believed that colonoscopy can be performed safely four to six weeks after the acute episode, and is particularly important in patients older than 40 years. In this case, the colonoscopy was performed after seven weeks and showed features compatible with appendicular mucocele.

Appendicular mucocele refers to the chronic transformation of the appendix into a mucus-filled sac and can be discovered accidentally or depicted in the clinical picture of AA [5]. Although a rare disease, surgical management of appendicular mucocele is mandatory due to the risk of malignant transformation and prevention of pseudomyxoma peritonei associated with mucocele rupture [5,6].

In our patient, the clinical suspicion of appendicular mucocele mandated appendectomy, which was performed without incident. Histology revealed a serrated adenoma and a parasitosis compatible with *E. vermicularis*.

There are four histologic types of appendiceal mucocele: retention cyst, mucosal hyperplasia, mucinous cystadenoma, and mucinous cystadenocarcinoma. Most benign mucoceles are due to mucinous adenoma, and in our case, a serrated adenoma was identified in histopathologic evaluation [5].

Serrated adenoma can produce mucus [12], but at the appendicular specimen, no mucus was found, opposite to the colonoscopy view, where mucus was protruding through the appendicular orifice. In fact, mucus production can be intermittent, such as in the present case.

AA induced by benign tumor obstruction is very uncommon. Adenomas are extremely rare appendiceal neoplasms, and the most common clinical presentation consists of incidental findings in the appendectomy specimen [4].

The serrated adenoma was located at the tip of the appendix and did not cause an obstruction. On a revision of 475 appendectomies for the clinical diagnosis of AA, a serrated adenoma was present in three cases (0.6%); unfortunately, it is not clear if these patients have histological criteria of AA [13].

Serrated adenomas of the appendix appear to be more aggressive lesions than their counterparts in the colon and rectum. Microsatellite instability is recognized in serrated adenoma as a molecular pathway for colorectal cancer. However, no further treatment is needed. The majority of these lesions are incidental findings on appendectomies or in autopsies [12].

According to the World Health Organization (WHO) Classification of Tumors of the Digestive System (2019), appendiceal serrated lesions are classified into three subtypes, namely, hyperplastic polyp, serrated lesion without dysplasia, and serrated lesion with dysplasia [14]. Any serrated polyp < 10 mm without dysplasia does not require endoscopic surveillance and should be referred to screening. Colonoscopy revaluation is indicated at 10 years if an organized screening program is not available.

Enterobiasis is the most common intestinal helminth infection worldwide [7]. The role of *E. vermicularis* in appendicitis is controversial and debatable. It is not clear whether the invading organism actually causes the inflammation or if the parasites are incidental findings in cases where inflammation is already present. In fact, the parasite is most frequently identified in appendices without pathologic findings and is rarely associated with the histological changes of AA [15,16].

Parasitic infections rarely cause a clinical picture of AA, especially in adults. In our case, the parasite was found in the lumen and not in the epithelium, although the histologic feature was that of AA, which was defined by the presence of neutrophil infiltrate in the muscular propria. In our patient, *Enterobius vermicularis* and infiltration of muscular propria with neutrophils were both present, which confirms AA [17]. Chronic inflammatory and eosinophilic infiltrates at the appendicular wall were also detected. Eosinophilic appendicular wall infiltration is common in AA [18]. Some authors argue that the presence of eosinophils is due to an allergic component in AA [1,2].

## Conclusions

The particularities of this AA case are unique, having an unusual presentation as an appendicular abscess and a colonoscopy compatible with an appendicular mucocele. A laparoscopic appendectomy was performed. Histology revealed a serrated adenoma, *E. vermicularis*, features of chronic inflammation, and AA. Adenoma is exceptionally associated with AA, and the relationship between *E. vermicularis* and AA is a rarity.
